# Thyroid Autoimmunity in the Context of Type 2 Diabetes Mellitus: Implications for Vitamin D

**DOI:** 10.1155/2015/710363

**Published:** 2015-05-19

**Authors:** Konstantinos Toulis, Xanthippi Tsekmekidou, Evangelos Potolidis, Triantafyllos Didangelos, Anna Gotzamani-Psarrakou, Pantelis Zebekakis, Michael Daniilidis, John Yovos, Kalliopi Kotsa

**Affiliations:** ^1^Diabetes Center, Department of Endocrinology and Metabolism, AHEPA University Hospital, 54636 Thessaloniki, Greece; ^2^First Propaedeutic Department of Internal Medicine, AHEPA University Hospital, 54636 Thessaloniki, Greece; ^3^Laboratory of Nuclear Medicine, AHEPA University Hospital, 54636 Thessaloniki, Greece; ^4^First Department of Medicine, AHEPA University Hospital, 54636 Thessaloniki, Greece

## Abstract

Vitamin D deficiency has been associated with both type 2 diabetes mellitus (T2DM) and autoimmune disorders. The association of vitamin D with T2DM and thyroid autoimmunity (TAI) has not been investigated. Thus, we aimed to explore the putative association between T2DM and thyroid autoimmunity (TAI) focusing on the role of 25-hydroxy-vitamin D (25(OH)D). Study population included 264 T2DM patients and 234 controls. To explore the potential association between 25(OH)D and thyroid autoimmunity while controlling for potential confounders—namely, age, gender, body mass index, and presence of T2DM—multivariate logistic regression analyses were undertaken. Patients with T2DM were younger (*P* < 0.001) and had significantly lower 25(OH)D levels (*P* < 0.001) and higher anti-TPO titers (*P* = 0.005). Multivariable logistic regression analyses suggested that T2DM and 25(OH)D levels were significantly associated with the presence of thyroid autoimmunity. In an elderly population of diabetic patients and controls with a high prevalence of vitamin D deficiency/insufficiency, a patient with T2DM was found to be 2.5 times more likely to have thyroid autoimmunity compared to a nondiabetic individual and the higher the serum 25(OH)D levels were, the higher this chance was.

## 1. Introduction

The association between type 2 diabetes mellitus (T2DM) and the presence of thyroid autoimmunity (TAI) has been a matter of debate. Although earlier reports dismissed any link between them [1–3], there is evidence to suggest an increased prevalence of TAI in patients with T2DM [4, 5]. Considering the discordant results, the direct association between TAI and newly identified hypothyroidism in the context of T2DM [6], and evidence implying a detrimental role for hypothyroidism in insulin sensitivity [7], it could be advocated that the association between T2DM and TAI requires further clarification from both the clinical and research perspectives.

Vitamin D, beyond its pivotal role in the regulation of calcium and phosphate homeostasis, emerges as a potentially important coregulator of both autoimmunity [8, 9] and insulin sensitivity [10, 11]. Vitamin D is considered to promote Th2 over Th1 immune response phenotype [12] acting through the vitamin D receptor- (VDR-) expressing immune cells [12, 13], while influencing both insulin secretion and sensitivity acting at the level of pancreatic beta-cells and muscle cells [10, 14].

In general, vitamin D deficiency or insufficiency is thought to be a predisposing factor to both autoimmune disorders and glucose intolerance. This preliminary evidence might provide the biological background to suggest that vitamin D could be involved in the putative association between T2DM and TAI; however, such hypothesis has not been addressed in the literature as yet.

To this end, we aimed to explore the association between T2DM and TAI focusing on the role of 25(OH)D in an ethnically homogenous, elderly population, using a cross-sectional, nested within case-control, study design.

## 2. Materials and Methods

Subjects with an established diagnosis of T2DM were consecutively recruited from the outpatient diabetes clinic of a tertiary reference hospital from December 2011 to March 2012. Community-dwelling individuals from the same region, in whom normal glycaemia was documented, both by fasting glucose (FPG) and glycated haemoglobin (HbA1c), were also recruited as controls during the same period. Controls were recruited from community centres providing social services to senior citizens (KAPI, Open Centres for the Protection of the Elderly). Current use of corticosteroids served as an exclusion criterion, since this could act as a confounder both at the levels of autoimmunity and glucose homeostasis. Subjects under vitamin D supplementation were also excluded from the study. At the day of the recruitment, a structured medical interview and a physical examination were performed in each subject, medical records were retrieved, and blood samples were drawn and stored at −80°C. Informed consent was provided and the study was conducted in accordance with the Declaration of Helsinki. Descriptive characteristics of the study population are summarized in Table 1.

### 2.1. Measurements

25-Hydroxy-vitamin D (25(OH)D), glycated haemoglobin (HbA1c), autoantibodies against thyroid peroxidase (TPOab) and thyroglobulin (TGab), and thyroid-stimulating hormone (TSH) were determined for each subject. Radioimmunoassays (DiaSorin, RIA) were performed according to the manufacturer's instructions for the measurements of serum 25(OH)D (intra-assay coefficient of variation (CV): 5.19%, interassay CV: 7.90%, and detection limit: 4 ng/mL) and thyroid parameters (TPOab intra-assay CV: 4.1%, interassay CV: 9.1%, and detection limit: 0.8 U/mL and TGab intra-assay CV: 2.9%, interassay CV: 11.6%, and detection limit: 6 U/mL). HbA1c measurements were performed by a standardized high performance liquid chromatography (HPLC) assay.

### 2.2. Definitions

A subject was included in the control group (subjects without diabetes) if FPG was less than 7.0 mmol/L (126 mg/dL) and HbA1c less than 48 mmol/mol (6.5%) and no use of diabetes medications was reported. A subject was designated as TAI positive if either TPOab or TGab was higher than 100 U/mL and as vitamin D deficient/insufficient if 25(OH)D was below 75 nmol/L (30 ng/mL). Presence of hypothyroidism was documented by a TSH value greater than 4.0 mU/L or thyroxine treatment.

### 2.3. Statistical Analysis

Continuous and dichotomous variables were described as mean (standard deviation) or *n* (%), respectively. Normality assumption was assessed by visual inspection of the distribution as well as the Kolmogorov-Smirnov test. Differences in means were explored using Mann-Whitney and chi-squared tests for continuous and dichotomous variables, respectively. To explore the potential association between 25(OH)D and thyroid autoimmunity while controlling for potential confounders, namely, age, gender, body mass index (BMI), and presence of T2DM, multivariate logistic regression analyses were undertaken in all study populations. Standardized values (z-scores) of the natural logarithms for all continuous variables were used. All analyses were undertaken within Stata 10.0.

## 3. Results 

**Figure 1:**
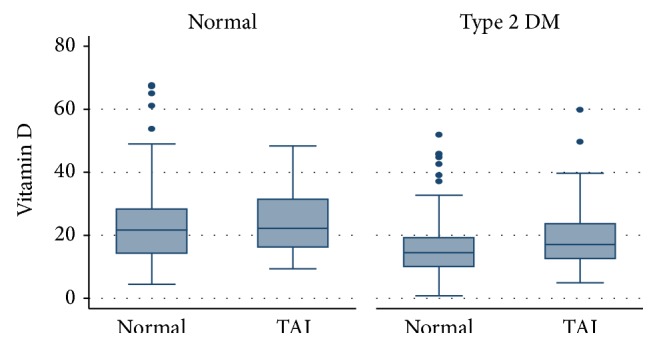
Box-plot representing serum 25-hydroxy-vitamin D levels by study group and thyroid autoimmunity status.

A total of 498 participants (264 patients with T2DM and 234 healthy controls) constituted the study population. Patients with T2DM were younger and more obese and had significantly lower 25(OH)D and higher TPOab titres compared to controls (Table 1). Prevalence of hypothyroidism was comparable between groups; however the prevalence of thyroid autoimmunity was approximately twofold greater in patients with T2DM compared to controls. Interestingly, the great majority of the study population (78%) was found to be 25(OH)D deficient or insufficient and the prevalence of vitamin D deficiency/insufficiency was significantly higher in patients with T2DM compared to controls. This finding was robust when a lower threshold for 25(OH)D (50 nmol/L (20 ng/mL), 65.5% versus 47% in patients with T2DM and controls, resp., and chi2 *P* value < 0.001) was applied. 25(OH)D levels by study group and TAI status are presented in Figure 1.

Multivariable logistic regression analyses adjusting for age, gender, and body mass index suggested that both the presence of T2DM (odds ratio (OR): 3.31, 95% confidence interval (CI): 1.58–6.90) and 25(OH)D levels (OR: 1.32, 95% CI: 1.01–1.72) were significantly associated with the presence of TAI (Table 2). Interpreting this finding in clinical terms, each unit increase in 25(OH)D levels (in standard deviations, corresponding to approximately 30 nmol/L (12 ng/mL) in the study population) is associated with a 30% increase (in odds) for TAI. 25(OH)D is depicted in ng/mL.

## 4. Discussion

In an elderly population of patients with T2DM and controls with a high prevalence of vitamin D deficiency/insufficiency, T2DM and 25(OH)D levels were significantly associated with TAI. The association between 25(OH)D and TAI was noted only in the presence of T2DM and, interestingly, it was found to be positive. Conflicting results have been reported in the literature regarding the association between T2DM and TAI [1–7]. These contradictory findings might either reflect T2DM phenotypic heterogeneity or result from the limited statistical power, multiple confounders, uncontrolled design, and ethnic heterogeneity in individual studies. We hypothesized that this inconsistency might imply a role for vitamin D in light of evidence suggesting its potential link to both thyroid autoimmunity and glucose intolerance individually. In specific, it has been reported that allelic variations within the VDR gene may mediate susceptibility to thyroid autoimmunity [15, 16]. Vitamin D insufficiency has been associated with thyroid autoimmunity [17], serum 25(OH)D levels were found to be significantly lower in patients with thyroid autoimmunity compared to controls [18, 19], and the severity of 25(OH)D deficiency correlated with thyroid antibody levels in both adult [18, 19] and children populations [20]. However, VDR knockout mice did not differ in parameters of thyrocyte function and morphology compared to wild-type controls [21], the prevalence of thyroid autoimmunity did not differ between subjects stratified on the basis of 25(OH)D levels [22], and vitamin D status was not associated with thyroid autoimmunity after controlling for sex and age [23]. Furthermore, euthyroid subjects with documented genetic susceptibility for developing thyroid autoimmunity did not differ in 25(OH)D levels compared to controls at baseline, which was also the case in those who finally developed de novo TPOAb compared to controls, questioning the role of vitamin D at least during the early stages of thyroid autoimmunity [24].

Similarly, a higher prevalence of vitamin D deficiency has been reported in the presence of T2DM [25] and an inverse association between circulating 25(OH)D levels and risk of incident T2DM was documented (each 10 ng/mL increment in 25(OH)D levels was associated with a 4% lower risk of T2DM) [26]. It should be noted though that this association has been recently questioned when taking into account genetic determinants [27] and that vitamin D3 supplementation had no beneficial effect on glycaemic indices in healthy overweight or obese women [28]. Collectively, it could be extrapolated that these data suggest that any role vitamin D might have in the putative association between thyroid autoimmunity and T2DM would be complex and certainly not self-evident. In fact, our findings seem to corroborate this notion. First, the association between thyroid autoimmunity and T2DM was documented adjusting for the major confounders; a subject with T2DM is 2.5 more likely (in odds) to have thyroid autoimmunity compared to a normal individual. Second, vitamin D levels are significantly associated with the presence of thyroid autoimmunity in subjects with T2DM, but not in controls. Third, it was shown that the higher the serum 25(OH)D levels are in these patients, the higher the chance was for thyroid autoimmunity. The latter finding is counterintuitive and of borderline statistical significance; therefore it requires further confirmation in subsequent studies. Of note, when 25(OH)D levels were modeled as a categorical variable, this association was not robust and no threshold effect was documented. Patients with T2DM have statistically significant higher percentage of thyroid autoimmunity compared to controls (Table 1). Of note, both groups have a high prevalence of vitamin D deficiency/insufficiency and the difference between the two groups is marginally statistically significant. On the other hand, it was found that TAI is more prevalent when the levels of vitamin D are higher. Vitamin D is one among many factors that could potentially modify thyroid autoimmunity. This discrepant finding should be attributed to the confounding effect of age, BMI, and gender, namely, all variables used in the multivariable regression analysis. This is why we undertook a regression analysis, adjusting for potential confounders. Any inferences were based on the findings of the latter analysis. In this model of regression analysis, both vitamin D levels and T2DM were included as covariates although associated. However, the degree of correlation between T2DM and 25(OH)D levels in our sample was not as high as to undermine the analysis. In any case, the theoretical risk of multicollinearity overinflates standard errors. Thus, there would be a risk of an insignificant finding. This was not the case in our study with regard to vitamin D.

Since the complex interplay between thyroid autoimmunity and vitamin D in the context of T2DM has not been explored to date, it might be premature to suggest an alternative hypothesis before further confirmation nor could it be substantiated on the basis of our data or study design. However, it might be intriguing to hypothesize that the effects noted in our study might be compatible with a tissue-specific model in the action of vitamin D on autoimmunity. Possible immunomodulatory effects of vitamin D include inhibition of proinflammatory activity of CD4+ Th1 cells and their production of cytokines (interleukin 2 (IL-2), interferon- (IFN-) *γ*, and tumor necrosis factor-*α* [29]).

In addition to its anti-inflammatory effects, vitamin D also promotes Th2 responses by enhancing IL-4, IL-5, and IL-10 production, thus promoting a more anti-inflammatory phenotype (Th2 state) of the T cell compartment over the inflammatory Th1 state [12]. On the other hand, the formation of thyroid autoantibodies by thyroid-infiltrating lymphocytes and blood lymphocytes has been found to be a distinct procedure. Although the intrathyroidal process is characterized by a shift towards Th1, the stimulus inducing the formation of thyroid autoantibodies by blood lymphocytes commonly shifts the balance towards Th2. Therefore, immune deviation towards Th2 has long been questioned as appropriate therapy for autoimmune thyroid disorders and has even been suggested that it “could project the patient from the frying pan into the fire” [30]. It is obvious that the above hypothesis requires further experimental confirmation.

Considering the observational nature of the evidence and, thus, the inappropriateness for causality inference, caution is advisable in the interpretation of the findings. Notably, the study findings should not be extrapolated to different populations with different baseline characteristics. Our sample demonstrated significant prevalence of vitamin D deficiency and a higher prevalence of thyroid autoimmunity in patients with T2DM compared to controls. Both of these sample characteristics are consistent with previous reports [31, 32], thus reassuring external validity concerns. It should also be noted that oral glucose tolerance test was not performed for the diagnosis of diabetes mellitus and, thus, a marginal misclassification of patients with diabetes as controls could not be excluded.

However, the impact of limitation on the study findings is probably minimal, since the discrimination between the diabetic and control groups was performed on the basis of two glycemic indices (fasting glucose and HbA1c measurements), which secured a clear distinction between groups. Similarly, the potential confounding effect of seasonal variation in serum 25(OH)D levels [33] is also expected to be minimal, since the collection of blood samples was performed during the same season (winter).

## 5. Conclusions

In summary, in an elderly population of patients with T2DM and controls with a high prevalence of vitamin D deficiency/insufficiency, it was shown that T2DM and vitamin D were associated with TAI. A patient with T2DM was 2.5 times more likely (in odds) to have thyroid autoimmunity compared to a normal individual and the higher the serum 25(OH)D levels were, the higher the chance for thyroid autoimmunity was.

## Figures and Tables

**Table 1:** Descriptive characteristics of the study population.

	Normal	Type 2 diabetes mellitus	*P*
*N*	234	264	
Male gender (female)	89 (38)	109 (41)	NS
Age (years)	72.2 (6.5)	67.6 (9.7)	0.0001
Body mass index (kg/m^2^)	30.6 (4.9)	31.6 (5.7)	0.032
Type 2 diabetes mellitus duration (years)	N/A	10.0 (8.4)	
Glycated haemoglobin (%)	4.7 (0.5)	7.1 (1.5)	0.0001
25-Hydroxy-vitamin D (ng/mL)	22.6 (12.6)	16.5 (10.4)	0.0001
Presence of vitamin D deficiency/insufficiency	172 (73.5%)	215 (81.4)	0.04
Thyroid peroxidase Ab (IU/mL)	60 (156)	90 (200)	0.005
Thyroglobulin Ab (IU/mL)	44 (131)	54 (136)	NS
Thyroid autoimmunity	18 (7.7)	38 (14.4)	0.018
Thyroid stimulating hormone (*μ*IU/mL)	1.95 (1.60)	2.25 (3.64)	NS
Hypothyroidism	8 (3.4)	11 (4.2)	NS

Data presented as mean (standard deviation) or *N* (%). *P* values refer to Mann-Whitney test or Pearson chi-square. NS: nonsignificant at the level of 0.05.

Abs: autoantibodies.

**Table 2:** Multivariable logistic regression using thyroid autoimmunity as the dependent variable.

Covariates	Odds ratio	95% CI	*P *
Type 2 diabetes mellitus	3.31	1.58–6.90	0.001
Gender	0.64	0.35–1.18	NS
Age	0.97	0.26–3.55	NS
Body mass index	1.24	0.89–1.72	NS
25-Hydroxy-vitamin D	1.32	1.01–1.72	0.047

CI: confidence interval.
